# Supplementary material for: Machine learning to extract muscle fascicle length changes from dynamic ultrasound images in real-time
Source: PLoS One. 2021 May 26;16(5):e0246611. doi: 10.1371/journal.pone.0246611 (PMC8153491; doi:10.1371/journal.pone.0246611)
Supplement: S1 File — (PDF) [file pone.0246611.s001.pdf]

S1 File. Supplementary Material

Title: Machine Learning to Extract Muscle Fascicle Length Changes from Dynamic Ultrasound Images in Real-Time

Authors: Luis G. Rosa, Jonathan S. Zia, Omer T. Inan, and Gregory S. Sawicki

I. Pre-Processing Steps:

1. *Crop:*
   Our crop consisted of directly limiting the matrix size with simple loops to focus on the soleus. For this, we manually measured the general area in which the soleus could be seen to obtain the cropping coordinates and kept these resulting matrix sizes constant for all images in each set (each 1400). Because the soleus moves throughout the task and the crop remains the same, in some tasks the crop may have captured adjacent features in addition to the soleus. Although this simple approach yielded results we could work with, we believe more robust cropping and feature extraction techniques have the potential of improving results.

2. *Down-sampling:*
   Using the "block_reduce" function from Scikit Image, we first sparsely tested different value combinations to input into the "block_size" parameter while maintaining default machine learning (ML) model parameters. Our inputs into "block_size" were of the form (#, #). After preliminary testing, the "heaviest" down-sampling value combinations seemed the most promising, and hence we chose 4 down-sampling rates accordingly where Rate 1 is the "heaviest" and Rate 4 the "lightest". Heavy down-sampling means smaller resulting matrix, while lighter means the matrix retains more of the original image information. We did our hyperparameter sweep across all direct and cross-subject tasks, training combinations, and ML models for all four down-sampling rates. We averaged all these results and chose the "best" down-sampling rate based on the same metrics described in the main text consisting of scaling RMSE to match 0 to 1 and them summing the inverse with correlation to obtain the best performer (**Supp Table 1**).

3. *Format:*
   To facilitate our matrix management, we used the "flatten" function to reduce the dimensionality of these matrices while keeping all the same information. We also changed data types as needed throughout the implementation code.

Averages across all models		
	CORR	***RMSE***
Down sampling Rate 1	0.59 ±0.33	2.97 ±2.68
Down sampling Rate 2	0.59 ±0.32	3.23 ±2.66
Down sampling Rate 3	0.57 ±0.31	3.30 ±2.52
Down sampling Rate 4	0.55 ±0.30	3.41 ±2.50

S1 Table. Results from averaging all direct and cross-subject tasks across all 5 ML models for each of the down-sampling rates.

II. Hyperparameter Optimization:

In an attempt to remain impartial and give each ML model the same chance of success, we looked for what parameters had the most impact on performance for each and performed our grid search accordingly. We made efforts to account for each model having a different number of parameters. A small amount of preliminary testing was done to choose the tested values. See **Supp Tables 2** for all non-default parameters used.

Model	***Parameter 1***		***Parameter 2***	
	Name	*Tested Values*	*Name*	*Tested Values*
Lasso	alpha	0.0000000001<br>0.0000001<br>0.00001<br>0.0001<br>0.001<br>0.01<br>0.1<br>1<br>10<br>100<br>1000<br>10000<br>1000000	max_iter	100000
Ridge	alpha	0.0000000001<br>0.0000001<br>0.00001<br>0.0001<br>0.001<br>0.01<br>0.1<br>1<br>10<br>100<br>1000<br>10000<br>1000000	max_iter	None<br>(No max)
LinearSVR<br>(Parameter 3 max_iter=100000)	epsilon	0.0000000005<br>0.0000005<br>0.00005<br>0.0005<br>0.005<br>0.05<br>0.5<br>0<br>5<br>50	C	0.1<br>0.5<br>1<br>10<br>100<br>1000<br>10000<br>100000<br>1000000<br>10000000

Model	***Parameter 1***		***Parameter 2***	
	Name	*Tested Values*	*Name*	*Tested Values*
SVM	gamma	0.0000000005<br>0.000000005<br>0.00000005<br>0.0000005<br>0.000005<br>0.00005<br>0.0005<br>0.005<br>0.05<br>0.5	C	0.1<br>0.5<br>1<br>10<br>100<br>1000<br>10000<br>100000<br>1000000<br>10000000
Random Forest	n_estimators	10<br>100<br>400<br>700<br>1000<br>1300<br>1600<br>1900<br>2200<br>2800	max_depth	None<br>(No max)

S2 Table. List of non-default parameters per model and the used values for testing each via grid search.

III. Real-Time:

Even though the paper focuses on our pseudo real-time implementation for testing and quantifying machine learning (ML) model capabilities, we have implemented our architecture in real-time. As evidence, we have included a short video of its preliminary performance. We have also included videos to show what the current standards look like (hand-tracking, UltraTrack) and a summary of our benchmarks for correlation, RMSE, and processing time (**Supp Table 3**).

Notes:

- Hand-tracking (**S1 Video**): We, as many other labs, hand-track within the UltraTrack architecture. Yet even though there are other ways of hand-tracking muscle fascicles, they all revolve around going frame-by-frame to ensure accuracy. Still, it is important to note that due to the common artifacts observed in B-mode ultrasound images, it is hard for any individual to be consistently accurate, and even harder for consistent measurements to be done between experimenters. [Kwah, et al. 2013, Van Hooren, et al. 2020]

- UltraTrack (**S2 Video**): The most basic form of UltraTrack tracking can be performed in just a few minutes (depending on trial length), yet these basic results are often filled with heavy drift. The key-frame-correction feature greatly helps mitigate this, yet its performance is tied to the user being able to identify these "key-frames" that are the same length through the trial. This becomes especially hard during dynamical tasks where there is no clear base length that one can go back to. Hence, results might greatly vary depending on experimenter skill, experience, and willingness to spend time finding these "key-frames".

- Real-time (**S3 Video**): It took 20-30s minutes on average to go from attaching the probe to the participant to having the ML model ready to measure in real-time after processing training data via UltraTrack. Our video shows both direct training of free ankle dorsi/plantar/flexion, and cross task training by tracking walking with the same training as for the free ankle (Note that free ankle was the worst performing for cross task, **Table 2c**). Note that we used the optimized SVM model. Our current setup runs out of a Gigabyte Aero 15 XA laptop with an Intel i7-9750H CPU, RTX 2070 GPU, and 144Hz refresh rate 1080p display. Because our real-time implementation relies on taking screenshots of the live B-mode ultrasound feed, it is hard to measure the latency accumulated between the ultrasound probe, transducer, the laptop processing, and the laptop screen. Nonetheless, as can be seen in the video, this accumulated latency remains very small (~0.02 seconds by our estimates). The observed output frequency is of around 50 Hz, and there is no filtering or smoothing of the output curve. Each output value is an isolated estimate of the image currently on screen. Note that if the different system components (ultrasound probe, computer, etc.) were optimized towards this application, performance could be improved. Hence, we hope this work encourages future developments.

	**Muscle Fascicle Length Tracking Options**		
	Hand-Tracked	***UltraTrack***	***Machine Learning***
Correlation, r	>0.95	~0.90	~0.70
RMSE	<1 mm	~4-5 mm	~3 mm
Processing Time (1400 frames)	~ Hours (4-6)	~ Minutes (20-40)	Real-Time

S3 Table. Comparison of Current Options for Measuring Muscle Fascicle Length from B-Mode Ultrasound Images
