# Supplementary material for: Machine learning to extract muscle fascicle length changes from dynamic ultrasound images in real-time
Source: PLoS One. 2021 May 26;16(5):e0246611. doi: 10.1371/journal.pone.0246611 (PMC8153491; doi:10.1371/journal.pone.0246611)
Supplement: Attachment — Submitted filename: Response to Reviewers.docx [file pone.0246611.s005.docx]

PONE-D-21-01984
Machine learning to extract muscle fascicle length changes from dynamic ultrasound images in real-time

RESPONSE TO REVIEWER COMMENTS

# Reviewer #1:

This paper aims to provide a benchmarking assessment of using several available machine learning programs to track soleus fascicle lengths during different functional movements, including calf raises and walking. The authors have made all data available and I believe this manuscript has broad impact in the fields of biomechanics and rehabilitation.

I do have a few comments for the authors to consider before final publication:

In lines 145-147 the authors mention that there was no movement of the heels or knees during constrained plantarflexion. However, it is extremely difficulty to provide a strong plantarflexor torque without movement in this position without the legs constrained (i.e. thighs fixed so knees cannot move). Was this the case? The images that show the different tasks don't look like this was the case. If so, please add.

Thank you for this observation and important point. In our protocol, subjects were instructed to push down without lifting their heels and not to push down as hard as possible. They were encouraged to hold their knees down with their hands if needed. We did not notice any ankle lift. As consequence, we understand that the resulting contractions are not expected to be from those harder to attain torques that might elicit heel lift. Given that the images and language are consistent with what we did, we made no changes. Should more protocol details be necessary, we would be happy to add a supplementary section on it. Please note that greater variability in subject task performance would only strengthen our claim of being able to track fascicles in cross conditions.

In lines 197-199 - it is possible that I misinterpreted this analysis, but as lower RMSE would be better, I think there may be an error in your description on adding the inverse of RMSE (to make lower values better). Wouldn't inverting RMSE make higher numbers better since the usual RMSE would indicate lower is better? This is a bit confusing in this region so clarification would be appreciated.

ORIGINAL LINES 196-199: “That is, we normalized the RMSE values by dividing absolute RMSE by the largest observed across participants and tasks (=~14 mm). Then, with both correlation and RMSE on the same scale, we simply added the inverse of the RMSE (to make lower values better) to the correlation value and selected the hyper parameters within each ML model that yielded the largest sum…”

The reviewer has correctly pointed out that this can be confusing and thanks to this comment we found a mistake in our language. What we did was not invert but find the difference of 1 – X where X is the scaled value. If we had inverted the relationship would have stayed the same, but RMSE values would have stopped being on the same scale as correlation. To help clarify we present an example with the original text and then the revised text:

Assuming we are comparing two parameters both with yielding correlation $r=0.75$, one with $RMSE=2$ and the other $RMSE=5$, where 2 is better since it is lower. Following the protocol described on LINES 196-197 “That is, we normalized the RMSE values by dividing absolute RMSE by the largest observed across participants and tasks (=~14 mm). we first have:

$$\frac{2}{14}= 0.143 \frac{5}{14}= 0.357$$

where the 0.143 from the 2 is still the smallest and preferred. We did this first step to have RMSE on the same 0 to 1 scale as correlation and that is why we say in LINE 197 “Then, with both correlation and RMSE on the same scale,”.

Continuing with LINES 197-199 “we simply added the inverse of the RMSE (to make lower values better) to the correlation value and selected the hyper parameters within each ML model that yielded the largest sum…” where because we wanted the largest sum, we actually took 1 – X and not the inverse to make smaller values better as follows:

$$1-0.143=0.857 1-0.357=0.643$$

Note, that if we had taken the inverse as the original text says we would have had the following:

$$\frac{1}{0.143}=6.993 \frac{1}{0.357}=2.801$$

In both cases the resulting numbers from the $RMSE=2$ condition are larger and hence better for that largest sum that we are looking for. Note again that the scaling is lost with the inverse but kept when using 1 – X.

As to complete the example with what we did, we had the sum of the correlation and modified RMSE as:

$$0.75+0.857=1.607 0.75+0.643=1.393$$

Where again the parameter with $RMSE=2$ ends up being the better one.

As a final comment, for our own sanity check we did go back to our code to confirm that this was the case and that there was no mistake in how we selected parameters.

UPDATED LINES 196-199: That is, we normalized the RMSE values by dividing absolute RMSE by the largest observed across participants and tasks (=~14 mm). Then, with both correlation and RMSE on the same scale, we simply added the difference of 1 – the adjusted RMSE (to make lower values better) to the correlation value and selected the hyper parameters within each ML model that yielded the largest sum

Figure 4 - is the 7 seconds of data represented here standardized to the same portion of the gait cycle? Was RMSE/correlations different in various parts of the gait cycle? As indicated in the discussion, it would be expected if the correlations were higher during a portion of the gait cycle with less noise introduced by gait contact. Is this the case?

These are good questions. The 7 seconds are the last 7 seconds of the representative subject’s walking trial. If you notice the grey line, that is the UltraTrack ground truth for that subject on that task and it is the same on all plots on this figure. The blue lines are the resulting estimates from the ML model on those 7 seconds after being trained on the walking data from other subjects. This lets us see how well does training the ML model on another “random” subject allow us to track these 7 seconds of the representative subject. We had the plot with grey and all blues together at first, but the separation we think helps visualize it all more clearly.

In the discussion we mention analyzing different parts of the gait cycle more as a potential future direction, but we did not run correlation analysis per gait phase. So, we have those same questions as you and would like to dedicate future research towards finding how gait phase and task might affect quality of tracking. Note that this would be an interesting discussion/finding not only for our protocol, but for the general ultrasound tracking methods.

# Reviewer #2:

In this study, the authors have investigated the machine learning to extract muscle fascicle length changes from dynamic ultrasound images in real time. The topic of the manuscript is interesting. However, there are some minor concerns that should be addressed before the article can be accepted for publication.

1.Please describe the purpose of the study more specifically.

ORIGINAL LINES 104-111: “In this paper we benchmark a hybrid framework that pairs ML+ functional ultrasound and can achieve real-time tracking of muscle fascicle length trajectories from live images of human muscle in dynamic conditions. We show B-mode ultrasound images of soleus muscle during different locomotion-relevant tasks can be fed to a number of different ML models which yield fascicle length estimates with low error and high correlation when compared to UltraTrack processed ground truth data. The resulting real-time, dynamic muscle imaging framework promises to not only speed up post hoc processing and analysis of ultrasound images of *in vivo* muscle contractile behavior, but also unlocks the possibility for novel muscle-in-the-loop applications that use muscle states to drive biofeedback training and/or assistive/rehabilitation devices [24].”

The reviewer is correct that we could have made this clearer and more direct throughout the paper. Our goal is to show that there is potential in training traditional ML models with ultrasound images to be able to track muscle fascicles in real-time, and that even with a protocol that takes ~20 minutes (from putting the probe on to having the ML trained and running live) results are “usable”. To clarify, we made some changes to the end of our introduction.

UPDATED LINES 104-112: In this paper, towards developing a protocol that allows real-time measurement of muscle fascicle length changes, we benchmark a hybrid framework that combines ML and functional ultrasound to obtain real-time length changes from live images of human muscle in dynamic conditions. We show B-mode ultrasound images of soleus muscle during different locomotion-relevant tasks being fed to a number of different ML models yielding fascicle length estimates with low error and high correlation when compared to UltraTrack processed ground truth data. The resulting real-time, dynamic muscle imaging framework promises to not only speed up post hoc processing and analysis of ultrasound images of *in vivo* muscle contractile behavior, but also unlocks the possibility for novel muscle-in-the-loop applications that use muscle states to drive biofeedback training and/or assistive/rehabilitation devices [24].

2.Please explain more about how the ultrasound images were obtained while the particitares rotate their ankle and walk task simultaneously?

Thanks for the comment. We are unsure of which part in the paper hinted towards the ankle rotation and walking tasks happening simultaneously. Note that all tasks happened separately with at least a minute of rest in between and that ultrasound images were collected the same way for all. To make this explicit, we updated the text as follows.

UPDATED LINES 143-154: “We selected five ankle movement tasks (**Fig. 1**) to capture kinetic and kinematic conditions spanning high and low muscle forces and high and low muscle displacements; an array that captures muscle states experienced during natural movements (*e.g.,* locomotion: stance phase = high force, low displacement and swing phase = low force high displacement) [25]. For every participant the tasks were performed in the same order with at least 1 minute of rest in between each as follows: a) *constrained ankle flexion*: while seated, participants performed push-release loading cycles with their foot against the ground and no movement of their heels or knees, b) *free* *ankle dorsi/plantar/flexion*: while seated, participants plantarflexed and dorsiflexed over a full range of motion with their foot in the air, c) *calf raises*: while weight-bearing, participants cyclically raised and lowered their heels as far as they could d) *walking*: participants walked at 1.5 m/s on a treadmill (Tuff Tread, Inc.), and e) a *mix*: participants were encouraged to move their foot as desired while staying in place, but alternating weight-bearing and foot in the air. For tasks a-c, participants were encouraged to practice the timing so that they could perform around ~7 cycles of each task during the 24 seconds (3-3.5 Hz). Participants were offered breaks to rest between tasks, yet none required them.”

3. In table 1 and 2, the parameter "CORR(r)" are calculated with other clinical data. Please describe how the values of "CORR(r)" are estimated with input data.

Thanks for the highlight and please let us know if there is a part or section of the manuscript indicating correlation was calculated with a different data set/clinical data as we are unsure. We can confirm that in all tables the correlation is calculated with our data and not with other clinical data but do let us know if we are missing something.

To calculate Pearson’s correlation we used the NumPy np.corrcoef() function. This function takes two arrays of the same length as input. We used the ground truth and the ML estimates as these two inputs and the function then goes and compares them to give us the resulting correlation score. Correlation values like those in table 1 and 2 are an average of correlation values as specified throughout the text. To make this more explicit, we added an extra sentence under the Model Evaluation section.

UPDATED LINES 220-231: “Using RMSE and Pearson’s Correlation Coefficient (*r*), we evaluated three potential muscle fascicle B-mode ultrasound imaging data *train-test* use cases: a) *direct training*: in which we trained the ML model and then tested it using data from the same participant and the same task (*e.g.,* train on participant #3 walking data to measure participant #3 during walking), b) *cross-participant training*: in which we trained the ML model with data from a different participant than the one being tested (*e.g.,* train on participant #6 walking data to measure participant #3 during walking), and c) *cross-task training:* in which we trained the ML model on data from the same participant but from a different task than the one being tested (*e.g.,* train on participant #3 using heel raises to measure participant #3 during walking). Note that we used the cross-task condition to measure only walking. We used the same train-test analysis protocol for direct, cross-participant and cross-task cases in which, of 1400 total image frames per condition, we used the first 1000 frames to train to ‘ground truth’, then the last 400 frames to test versus ‘ground truth’. Testing against ground truth meaning we calculated the correlation and RMSE values between the 400 ground truth frames and the ML-model-predicted 400 frames.”

4.In table 2, what the possible reason for lower values of "CORR(r)" for cross-task training? And what's those values mean in the red color in table 1and 2?

Why we see lower cross-task correlation values is a very valid question which we address in section 3.2, specifically on lines 296-305 copied below.

LINES 296-305: “From cross *task* training we obtained an average correlation of 0.22 ±0.18 and average RMSE of 2.36 ±1.03mm when measuring walking data after training our SVM ML model with data from the other movement tasks (**Table 2c**). These correlation values are lower than direct or cross-participant training since each task covers a different combination of ankle joint range of motion, soleus muscle loading, and overall noise. With under 20 seconds of training data (1000 frames), it was challenging to generalize training and make accurate muscle fascicle length measurements in walking. However, we observe once again that despite low formal correlations (<0.6) our ML based tracking still generates useable data that captures qualitative features of ground truth quite well. (**Fig. 5**). After a closer look at our data, we observe that when compared to cross-participant or direct training, correlation averages for cross-task training are much lower due to a more frequent lack of correlation. Thus, despite potential convenience, there is a larger risk of poorer muscle fascicle length measurements when employing cross-task training vs. the other approaches (**Table 2**).”

Values in red on the different tables indicate the highest correlation and lowest RMSE to facilitate seeing the best performer in each category. We believe this is beneficial for the reader, yet we are aware that PLOS ONE table format requirements might not even allow color in tables, hence we removed the color red.

5.It was mentioned six participants voluntarily participated in this experiment. Can the authors explain that the database is enough for training machine learning?

This is a very good point. All the results shown are from training with only one participant and not a combination of any of the 6 subjects. This helped us show how well the simplest approach performs. Still, we can assume more robust training and implementation of more advanced machine learning techniques could improve performance. Even if a performance increase might be unnecessary for certain applications, we have updated our discussion text to properly address this.

UPDATED LINES 410-422: It is important to note that our approach to optimizing the ML-based estimates of muscle fascicle length changes from B-mode images was not exhaustive, but instead represents a first step toward the long-term goal of developing optimized muscle-level feedback systems for augmented movement. For example, the amount of data we used for training, 1 subject and 1 condition (1000 frames), is minimal in comparison to what most traditional convolutional neural networks use, yet it shows how well a simple lab-applicable approach performs. Some ways of improving our ML model based predictions include: 1) improving ground truth quality by considering more accurate alternatives to UltraTrack, 2) optimizing the size and image quality of the training data set 3) implementing real time filtering and signal processing techniques (*e.g.,* Kalman, etc.), 4) using more capable imaging hardware (*e.g.,* increasing ultrasound sampling frequency in time, reducing latency, etc.), 5) optimizing feature level aspects of the image processing through time (*e.g.,* increasing resolution at a specific gait phase), 6) devising movement tasks that can generate training sets that robustly generalize for the intended application, and 7) improving the pre-processing phase to include more robust feature extraction.

6.Which ML model is optimal by the results in this study?

We obtained the best performance with SVM.

See lines from original text below:

LINES 234-238: “In this paper, for the first time, we were able to demonstrate the feasibility of ML algorithms for obtaining accurate muscle fascicle length measurements from B-Mode ultrasound images in real-time (**S1 File III, S3 Video**). After optimizing each ML model (Lasso, Ridge, Linear SVR, SVM, Random Forest) to simultaneously maximize Pearson’s correlation, *r* and minimize RMSE with equal weighting across all movement tasks, we obtained the best performance using SVM for both direct and cross participant training scenarios (**Table 1**).”

# Reviewer #3:

The authors attempted to use machine learning (ML) models to record muscle fascicle lengths in real-time. The current result showed that the performance of support vector machine (SVM) model was the best with an acceptable r and RMSE, indicating that it is possible to track soleus muscle fascicle with ML models. However, there are still some issues that need to be clarified.

MAJOR COMMENT
Introduction
I was wondering why this study chose soleus as the target muscle. Could the authors explain why measuring soleus muscle fascicle length is important? For me, gastrocnemius may be more important than soleus in functional activities.

This is a very valid concern. For us, based on the existing links between soleus and metabolic cost and our future interests on reducing this in real-time, we decided to show our work with soleus. It is understandable that some might argue gastrocnemius would have had higher impact and we are not denying this yet do note that the same or very similar results are expected for muscles with fascicles of similar lengths like gastrocnemius even if not shown or discussed in this paper.

For more on why we are interested in soleus see:

[26] Nuckols RW, Dick TJM, Beck ON, Sawicki GS. Ultrasound imaging links soleus muscle neuromechanics and energetics during human walking with elastic ankle exoskeletons. Sci Rep. 2020;10: 1–15. doi:10.1038/s41598-020-60360-4

Methodology
First, how did you stabilize the ultrasound transducer on the muscle belly in performing dynamic tasks? There are various equipment or devices that stabilize the probe during dynamic movement in the past studies. Please elaborate this experimental setting in the method.

Thanks for pointing this out. We used 3M Vetrap Bandaging Tape to secure the transducer. This is a soft self-adhesive tape which we have found works well to secure the probe in place even during dynamic movements thanks to how much pressure it can hold. We have updated the text to include this.

UPDATED LINES 118-120: “B-mode ultrasound data were collected for five 24-second tasks (**Fig.1**) with the ultrasound probe (Telemed, ArtUs EXT-1H, LV8-5N60-A2 probe) wrapped to the right calf with 3M Vetrap Bandaging Tape to obtain images of soleus muscle fascicles (**Fig. 2A**).”

Second, why did you ask participants to perform each movement in the same order? Did that affect the outcomes?

This a valid concern. We were not expecting any effects from this and did not observe anything that would have indicated otherwise. The only way we could visualize any significant changes would be if under very strenuous tasks which significantly affect the muscle fascicle length ranges the subject can reach, since that would limit the length ranges we train our models on. In such a case, testing would be required. Yet given we only collected ~24 seconds of data per task and that subjects rested in between each, we were not expecting any effects or changes from task order.

Third, I did not understand the reason to select the last part of data as ground-truth rather than the random samples. Could you explain the explanation more? Why could this approach simulate the real-time measurement settings?

Thanks for the comment. We selected a continuous section of the data as ground truth and for testing since it would allow us to see the changes in time and would allow us to compare to expected values and curve shapes. Visual cohesion of data is important to us given potential real-time applications which could include visual biofeedback, and more. Furthermore, it emulates collecting a certain amount of data, using it for training, and then looking at the resulting real-time results. It aims to not only validate the models, but the lab protocol implementation as well.

Last, I did not understand how you calculated Pearson correlation coefficient. What 2 variables did you want to develop the correlation? How did you derive multiple r values to average them?

This reviewer was not the only one to comment on this, hence we realize it might not be as clear as we thought. To calculate correlation we used the NumPy np.corrcoef() function. This function takes two arrays of the same length as input. We used the ground truth and the ML estimates as these two inputs and the function then goes and compares them to give us the resulting correlation value. This correlation value represents how well the changes in length match. Correlation value averages average all the corresponding sub-components, for example, in table 1a, we obtained the correlation values for each of the models by averaging the correlations obtained for every subject and every task with direct training for that model. We updated the text in two sections.

UPDATED LINES 235-252: In this paper, for the first time, we were able to demonstrate the feasibility of ML algorithms for obtaining accurate muscle fascicle length measurements from B-Mode ultrasound images in real-time (**S1 File III, S3 Video**). After optimizing each ML model (Lasso, Ridge, Linear SVR, SVM, Random Forest) to simultaneously maximize Pearson’s correlation, *r* and minimize RMSE with equal weighting across all movement tasks, we obtained the best performance using SVM for both direct and cross participant training scenarios (**Table 1**). Note that we did not implement a neural network since after preliminary testing non-linear models like Random Forest, we did not see a significant performance increase. SVM yielded an average correlation (*r*) of 0.70 ±0.34 with an average RMSE of 2.86 ±2.55mm when optimized for all direct training conditions (**Table 1a**) and yielded an average correlation of 0.65 ±0.35 with an average RMSE of 3.28 ±2.64mm when optimized for all cross-participant movement tasks (**Table 1b**). Note that correlation value averages in all Tables are averaging all the corresponding sub-components (e.g., in **Table 1a** we obtained correlation values for each of the models by averaging the correlations obtained for every subject and every task with direct training for that model). These results show moderately strong correlation values comparable to previous reports documenting muscle fascicle length tracking algorithms. Indeed, our result demonstrates RMSE of ~3mm or ~6% of the median fascicle length (=~50mm) for most trials, which is in line with the 10% RMSE seen in related studies [6,13,14]. It is important to note that we obtained similarly strong results from all five ML models, indicating that, if properly tuned, many ML models have potential to generate accurate tracking of muscle fascicle length from B-mode images without requiring deep learning. Given its overall best performance, we focus our analysis on direct, cross-participant, and cross-task training for the optimized SVM ML model *only*.

UPDATED LINES 220-231: “Using RMSE and Pearson’s Correlation Coefficient (*r*), we evaluated three potential muscle fascicle B-mode ultrasound imaging data *train-test* use cases: a) *direct training*: in which we trained the ML model and then tested it using data from the same participant and the same task (*e.g.,* train on participant #3 walking data to measure participant #3 during walking), b) *cross-participant training*: in which we trained the ML model with data from a different participant than the one being tested (*e.g.,* train on participant #6 walking data to measure participant #3 during walking), and c) *cross-task training:* in which we trained the ML model on data from the same participant but from a different task than the one being tested (*e.g.,* train on participant #3 using heel raises to measure participant #3 during walking). Note that we used the cross-task condition to measure only walking. We used the same train-test analysis protocol for direct, cross-participant and cross-task cases in which, of 1400 total image frames per condition, we used the first 1000 frames to train to ‘ground truth’, then the last 400 frames to test versus ‘ground truth’. Testing against ground truth meaning we calculated the correlation and RMSE values between the 400 ground truth frames and the ML-model-predicted 400 frames.”

Results and Discussion
Could you separate the results from discussion? I still think it would be better to make 2 sections apart.

Thanks for the suggestion. This is something we had conversations about. The goal behind the paper is not to show that we have the highest correlation and that the numbers are the best in the field, but instead to demonstrate the value behind them and how they show ML’s viability as a tool for measuring fascicle lengths in real-time. Hence, having results and discussion together does better justice to the paper and what it is trying to convey.

In addition, I was curious why the SVM was the best model. Was there any possible reason?

Thanks for the highlight. The ‘rbf’ kernel we used on the SVM model is non-linear which helps handle variability. Similarly, the mathematical formulation behind SVM helps it handle image data well. Compared to other simpler models (Ridge, Lasso, etc, which are linear), the higher performance was expected.

The bigger surprise came from such similar performance with simple models and a lower performance from the non-linear Random Forest model. This could be a result of the models’ interaction with the down-sampled data and the parameter optimization. Do note that we do not discard the possibility of further improving the performance of any of these models with a more comprehensive optimization process, which might change what the optimal model is.

MINOR COMMENT
Line 240: What does “moderately strong correlation” mean? I consider “moderate to strong” might be a better expression.

We have updated the text accordingly.

UPDATED LINES 246-247: These results show moderate to strong correlation values comparable to previous reports documenting muscle fascicle length tracking algorithms.

Line 383: “For example, it may be ‘that’ in…” What did “that” indicate in this sentence?

LINE 383: “For example, it may be that in some applications, accuracy is paramount only during a certain phase of the movement, bringing into focus a new set of factors in the image processing pipeline that will need to be optimized.”

After some confirmation from a grammatical engine, we have that it is being used as a pronoun. The phrase “it may be that” could be removed, yet it helps show that this is still uncharted territory about which we can only hypothesize.

Line 404-406: “Furthermore, feeding ultrasound images…robustness and/or resolution.” Could you elaborate more why feeding data from other devices that assess muscle quality could improve the measurement robustness and resolution? In my opinion, these devices record different qualities of muscle, so they provide different information regarding muscle.

We agree with your opinion. The theory is that if we had these different qualities of the muscle, that would mean more information about what the subject is doing, what the motions are, etc., which again, in theory, means we could better estimate expected muscle lengths changes and hence, with an appropriate model, we could improve our ML training by having additional features to fill potential gaps.

All tables: All tables need bottom border. And please put footnotes to explain CORR because r is not unit.

Thanks for catching that. Bottom border was added to all tables and an explanation with the title to clarify correlation is unitless and how averages were obtained.
